# Case Report: The unique case of flexible intramedullary nailing of pediatric radius complicated with temporary radial nerve’s motor branch damage

**DOI:** 10.3389/fped.2023.1325459

**Published:** 2024-01-05

**Authors:** Łukasz Wiktor, Ryszard Tomaszewski

**Affiliations:** ^1^ Department of Trauma and Orthopaedic Surgery, Upper Silesian Children’s Health Centre, Katowice, Poland; ^2^ Department of Trauma and Orthopedic Surgery, ZSM Hospital, Chorzów, Poland; ^3^ Faculty of Science and Technology, Institute of Biomedical Engineering, University of Silesia in Katowice, Katowice, Poland

**Keywords:** children, forearm shaft fracture, radial nerve injury, elastic-stable intramedullary nailing, motor branch of the radial nerve

## Abstract

This study reported a case of radius flexible intramedullary nailing complicated by temporary paralysis of the posterior interosseous nerve due to compression of the ESIN on the nerve in an 8-year-old boy. The nerve damage resulted from an essential misconception at the surgery. Despite bad decisions made during qualifications and the procedure undertaken, restoring the nerve function, and gaining satisfactory functional fracture recovery was possible. Although it is generally acknowledged to perform retrograde flexible intramedullary nailing from the level of the distal radial metaphysis, the presentation of our case aims to emphasize the real risk of damage to the motor branch of the radial nerve when approaching the proximal metaphysis.

## Introduction

Fractures of the forearm shafts in children are relatively common and constitute approximately 6% of all pediatric fractures (1). In children under ten, anatomical reduction of fractures is unnecessary because the potential for bone remodeling at the growth plate level allows for gradual correction of residual displacements (2). At this age, treatment with an arm cast is considered a gold standard. For older children, whose potential for spontaneous correction decreases with age, anatomical fracture reduction is essential to acquire a good functional effect (3, 4). The need for anatomical reduction and relatively frequent complications related to conservative treatment, including secondary displacements requiring additional procedures and inaccurate bone healing, in 1970 led to the development of elastic stable intramedullary nailing (ESIN) by the Jean Prevot and Paul Metaizeau at the Children's Hospital of Nancy in France (5, 6). Alternative methods of treating forearm fractures include open reduction with internal plate fixation, external fixation, or percutaneous Kirschner wire stabilization with the application of an arm cast (7, 8).

ESIN is a minimally invasive technique that allows obtaining anatomical fracture alignment to ensure proper bone union and, therefore, has been established as “state-of-the-art” for unstable forearm fracture treatment (9–11).

This study aimed to report a fracture of the distal third of the radius treated by anterograde intramedullary nailing associated with transient posterior interosseous nerve (PIN) palsy.

The PIN is a motor branch of the radial nerve that originates at the radiohumeral joint line. It runs under the supinator muscle at the arcade of Frohse divides for sub-branches, which are responsible for the innervation of the extensor muscles (estensor digitorum communis; extensor indicis proprius; extensor pollicis brevis and longus; abductor pollicis longus; extensor digiti minimi; supinator and extensor carpi ulnaris).

## Case report

**Figure 1:**
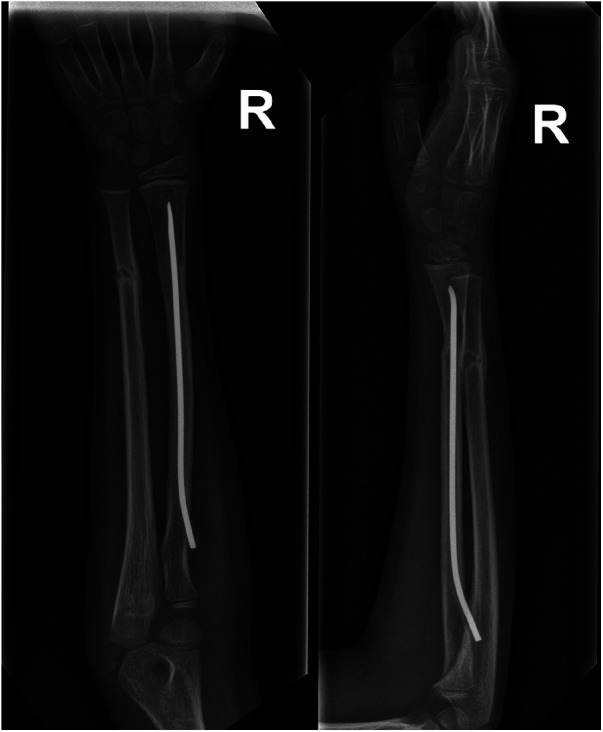
Seven weeks after initial surgery, a follow-up x-ray showed progressive bone union with proper bone alignment. In the radius, a flexible intramedullary nail inserted from the proximal metaphysis.

**Figure 2:**
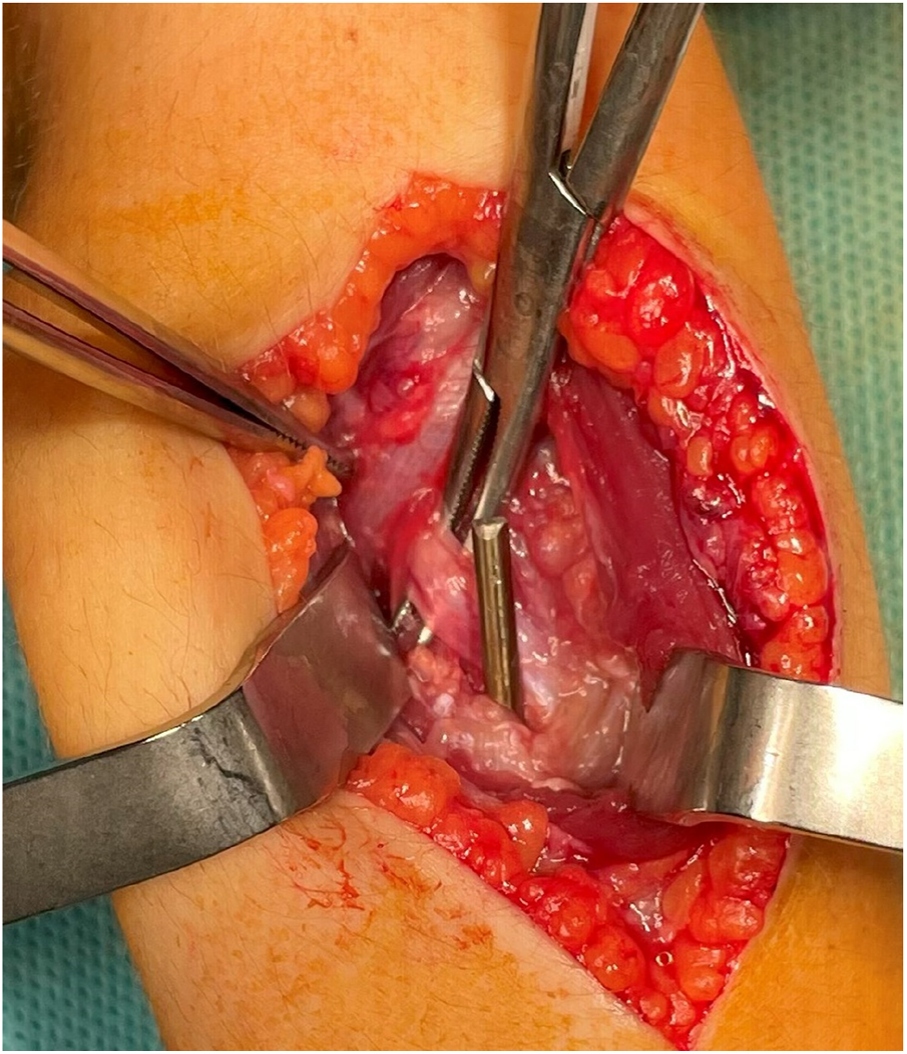
The intraoperative figure shows the titanium nail's end and its direct conflict with the deep branch of the radial nerve (nerve on the Pean clamp).

**Figure 3:**
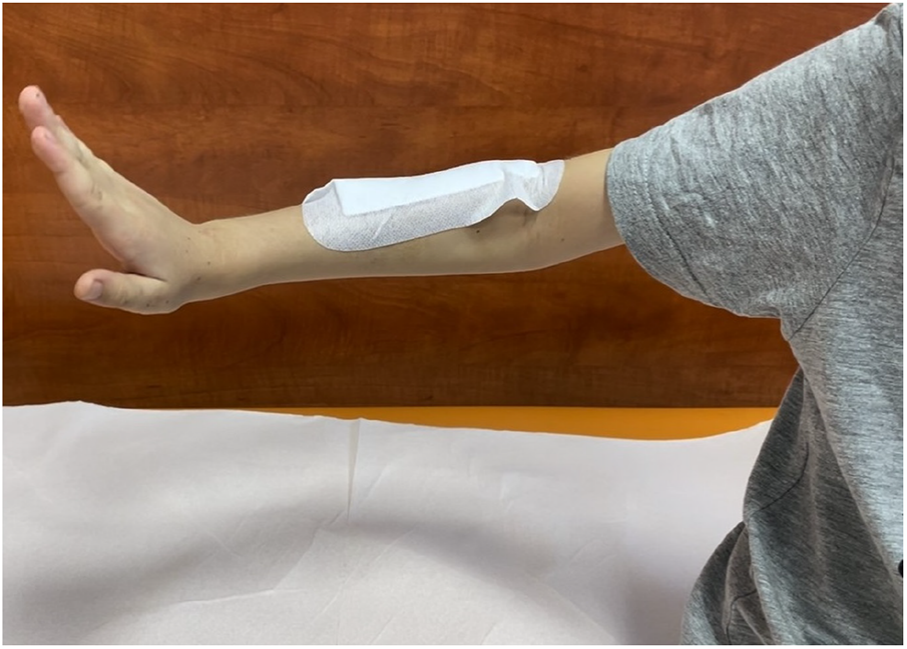
Figure showing full active extension of the wrist and fingers during the follow-up visit, two weeks after the surgical procedure.

A 8.5-year-old boy was admitted at our Trauma Center after he was treated elsewhere for a fracture of the distal third of radius. The patient also had type 1 diabetes that had been under treatment for about a year. According to the documentation, surgical treatment consisted of a closed reduction with ESIN of the radius. Due to the satisfactory alignment of the ulna fracture, internal fixation was abandoned. The surgery and the postoperative period were without any complications. After surgery, the upper limb was immobilized in an arm cast with the forearm in supination. After approximately five weeks, arm cast was discontinued, and the patient was instructed on gradual motor improvement and was referred to outpatient rehabilitation. About a week after starting the forearm exercises, there were gradually increasing symptoms of posterior interosseous nerve palsy, displayed by wrist drop and finger extension deficit. Despite the motor loss, the patient manifested no skin sensation disturbances. A precise neurological examination during the first visit to our Center is presented in Table 1. Muscle strength was assessed according to the British Medical Research Council Scale Table 2. A follow-up x-ray (Figure 1) showed progressive bone union with proper alignment. The titanium rod was inserted into the radius from the level of the proximal radial metaphysis. Due to anterograde stabilization, symptoms of radial nerve palsy were combined with the potential of iatrogenic radial nerve damage. The patient was qualified for urgent hardware removal. The operating team consisted of an orthopedist and a neurosurgeon. The surgical approach was widened to safely reach the nail and explore the deep branch of the radial nerve. Intraoperative figures show the end of the titanium rod and the radial nerve wrapped around the implant. The nerve-rod conflict was confirmed, involving stretching the nerve during the pronation movement of the forearm. After cautious implant removal, the correct nerve function was confirmed by intraoperative electrostimulation of the radial deep branch. The postoperative period was uneventful. During a follow-up visit two weeks after the procedure, the sutures were removed, and the function of the radial nerve was assessed similarly. A detailed neurological examination is shown in Table 3. The full active wrist extension is shown in Figure 2. A follow-up visit to the orthopedic outpatient three months after the surgery confirmed an excellent functional effect with a full range of motion in the elbow, wrist, and hand, Figure 3. The normal function of the radial nerve was approved.

**Table 1:** Neurological examination results during the first visit.

Wrist extension	Fingers extension	Thumb extension	Active wrist extension strength (BMRC)	Active finger extension (BMRC)	Active thumb extension (BMRC)	Skin sensations disturbances
Slight movement	Slight movement	Slight movement	M1	M1	M1	No deficit

**Table 2:** British medical research council muscle power scale (12).

Grade	Muscle power
0	No contraction
1	Flicker or trace of contraction
2	Active movement, with gravity eliminated
3	Active movement, against gravity
4	Active movement, against gravity and resistance
5	Normal power

**Table 3:** Neurological examination results during the follow-up visit, two weeks after the surgical procedure.

Wrist extension	Fingers extension	Thumb extension	Active wrist extension strength (BMRC)	Active finger extension (BMRC)	Active thumb extension (BMRC)	Skin sensations disturbances
No deficit	No deficit	Slight movement	M5	M5	M5	No deficit

## Discussion

Currently, closed reduction combined with elastic-stable intramedullary nailing (ESIN) is the preferred method of treating forearm diaphyseal fractures in children and adolescents if surgical treatment is required (displaced and/or unstable fractures). It is a relatively simple method, but the surgeon should be aware of the indications and follow the method's basic principles. Most operative failures occur by neglecting the crucial biomechanical principles and technical errors. The most common complications observed after ESIN include superficial wound infections, pseudarthrosis, delayed union, malunion, loss of correction—most commonly seen in the distal third, refracture, osteomyelitis, tendon rupture, forearm stiffness, and lesion of the superficial radial nerve (13–15). Lesions of the superficial radial nerve are a common complication related to ESIN, and they occur in primary surgery as well as at the time of material removal at a similar rate (9, 14, 16). The reason for damage to the sensory branches of the radial nerve is that the nerve splits into diverse branches at the surgical approach, typically at the distal and lateral aspect of the radial metaphysis. Insertion through Lister's tubercle (dorsal entry point) is related to a lower risk of nerve damage (17). It offers more versatile nail manipulation and is an alternative for distal 1/3 fractures of the radius. However, it is associated with a greater secondary extensor tendon injury risk. A sufficient approach (1,5–2 cm) and careful blunt subcutaneous preparation are recommended not to hurt the superficial radial nerve and the cephalic vein. This is especially important during material removal procedures because scarring obstructs the identification of the nerves and superficial vessels. In our particular case, the reason for damage to the radial nerve's deep branch was a cardinal mistake in the surgical technique involving the introduction of an elastic nail through a mini approach (approximately 1 cm) from the proximal end of the radius. The most popular surgical approaches to the proximal radius include the volar approach, described by Henry, and the dorsolateral approach, detailed by Thompson (18, 19). The surgeon operating must be aware that detaching the supinator muscle from bone requires protection of the radial nerve's motor branch. Fractures of the distal third of the forearm are undoubtedly demanding and constitute a questionable indication for ESIN. If the nail insertion point is located radially and the nail is not correctly pre-bent, obtaining proper bone alignment may be difficult. This may be why the orthopedist responsible for closed reduction badly inserted the nail from the side of the longer bone fragment. It is worth emphasizing that the knowledge of a method's possible failures and complications allows its correct application. While reviewing the available literature relating the results and possible complications of forearm ESIN, we did not find a similar case in which damage to the motor branch of the radial nerve was described.

## Conclusions

1. Closed reduction combined with elastic-stable intramedullary nailing (ESIN) is the preferred method of treating forearm diaphyseal fractures in children and adolescents.
2. A sufficient surgical approach (1,5–2 cm) at the distal aspect of the radial metaphysis is recommended to avoid damage to the superficial radial nerve branches and growth plate.
3. Anterograde introduction of an elastic nail from the proximal end of the radius is at high risk of iatrogenic posterior interosseous nerve injury.

## Data Availability

The original contributions presented in the study are included in the article/Supplementary Material, further inquiries can be directed to the corresponding author.
